# Experiences of Health Care Interactions Among Older Adults in Geropsychiatric Treatment Following Self-Harm

**DOI:** 10.1093/geroni/igaf122.1563

**Published:** 2025-12-31

**Authors:** Maja Sjöberg, Sara Hed, Stefan Wiktorsson, Anne Ingeborg Berg, Jennifer Strand, Sabrina Doering, Margda Waern

**Affiliations:** University of Gothenburg, Sahlgrenska University Hospital, Gothenburg, Vastra Gotaland, Sweden; University of Gothenburg, Gothenburg, Vastra Gotaland, Sweden; University of Gothenburg, Gothenburg, Vastra Gotaland, Sweden; University of Gothenburg, Gothenburg, Vastra Gotaland, Sweden; University of Gothenburg, Gothenburg, Vastra Gotaland, Sweden; University of Gothenburg, Gothenburg, Vastra Gotaland, Sweden; University of Gothenburg, Gothenburg, Vastra Gotaland, Sweden

## Abstract

Physical illness and functional disability are common in older adult populations and strongly linked to suicidal behavior. The aim was to explore how older adults who engaged in self-harm experienced their interactions with healthcare providers. Two separate semi-structured interviews were conducted with each participant. Transcripts were analyzed using Interpretative Phenomenological Analysis. The participants were recruited among consecutive Swedish-speaking patients in outpatient treatment following self-harm within the last 3-36 months. Exclusion criteria were personality disorder, ongoing psychosis, aphasia, delirium, clinical dementia, or Montreal Cognitive Assessment score indicating moderate/severe cognitive impairment. Out of 22 eligible, nine accepted participation (4 women and 5 men, age range 71-92 years). Prior to their engagement in self-harm, all had their main care contact in primary care and all but one were on antidepressants. Participants described interactions with health care services that amplified their feelings of alienation, loneliness, worthlessness and self-stigma. Difficulties accessing care increased their sense of powerlessness. Some participants were cognizant of their mental health needs but experienced obstacles that hindered them from managing their illness. These situations increased frustration and hopelessness and contributed to the development of suicidal behavior. On the other hand, feeling listened to in trustful and validating relationships helped restore self-respect and agency, and fostered engagement in their individual suicide preventative strategies. Finding can inform clinical approaches to the care of older adults with symptoms of common mental disorders. Exploring experiences of care interactions before and after suicidal acts across different clinical settings and cultures could be areas for future research.

